# Supplementary material for: Observation of coherent delocalized phonon-like modes in DNA under physiological conditions
Source: Nat Commun. 2016 Jun 1;7:11799. doi: 10.1038/ncomms11799 (PMC4895446; doi:10.1038/ncomms11799)
Supplement: Supplementary Information — Supplementary Figures 1-9, Supplementary Tables 1-4, Supplementary Notes 1-5 and Supplementary References [file ncomms11799-s1.pdf]

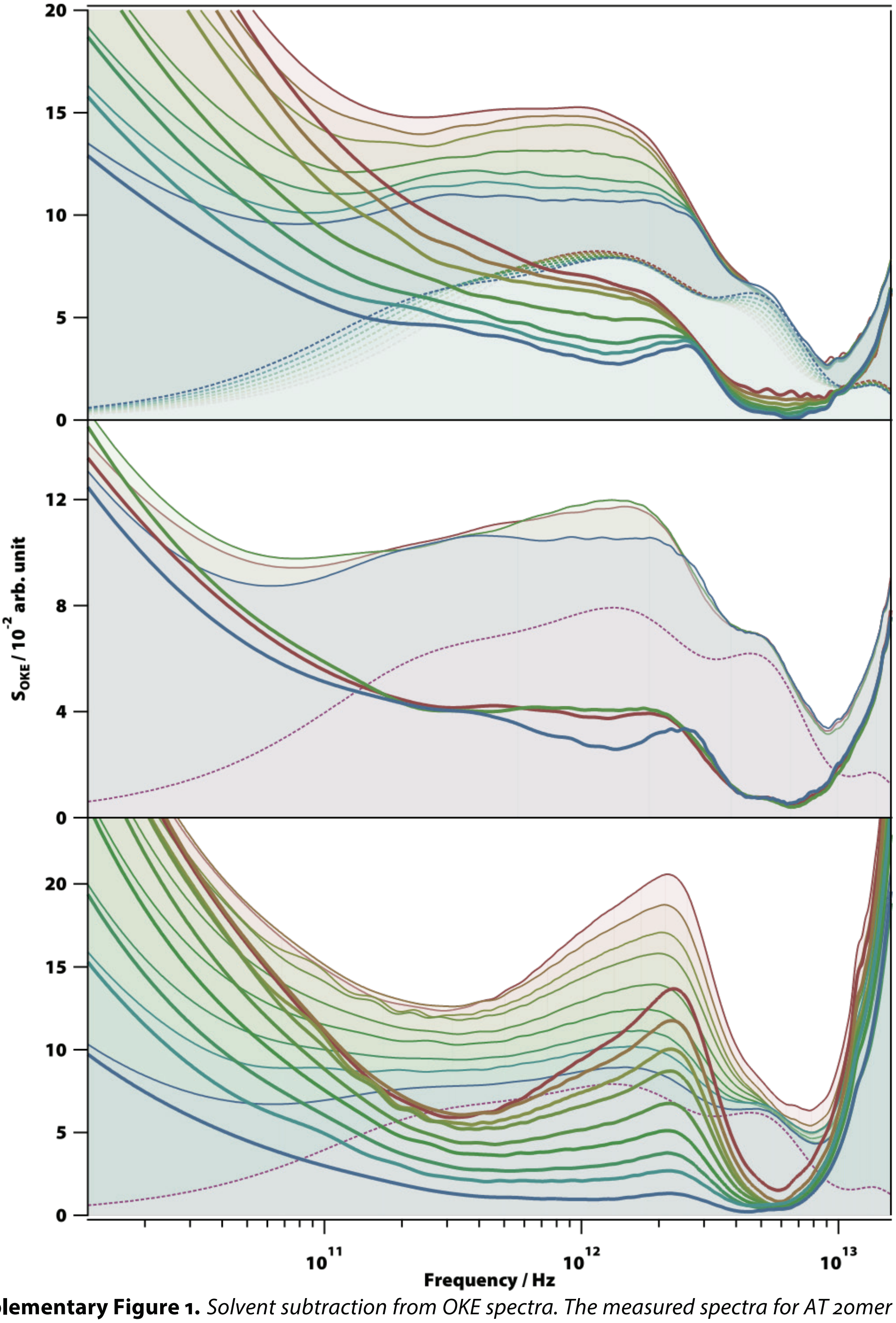

Supplementary Figure 1. *Solvent subtraction from OKE spectra. The measured spectra for AT 20mer (top), complementary oligomers (middle) and CMP (bottom) are represented with filled lines. To obtain the spectra of the solvated nucleic acids shown in figures 1, 2 and 3 (thick lines), water spectra (dashed lines) were subtracted from the measured spectra.*

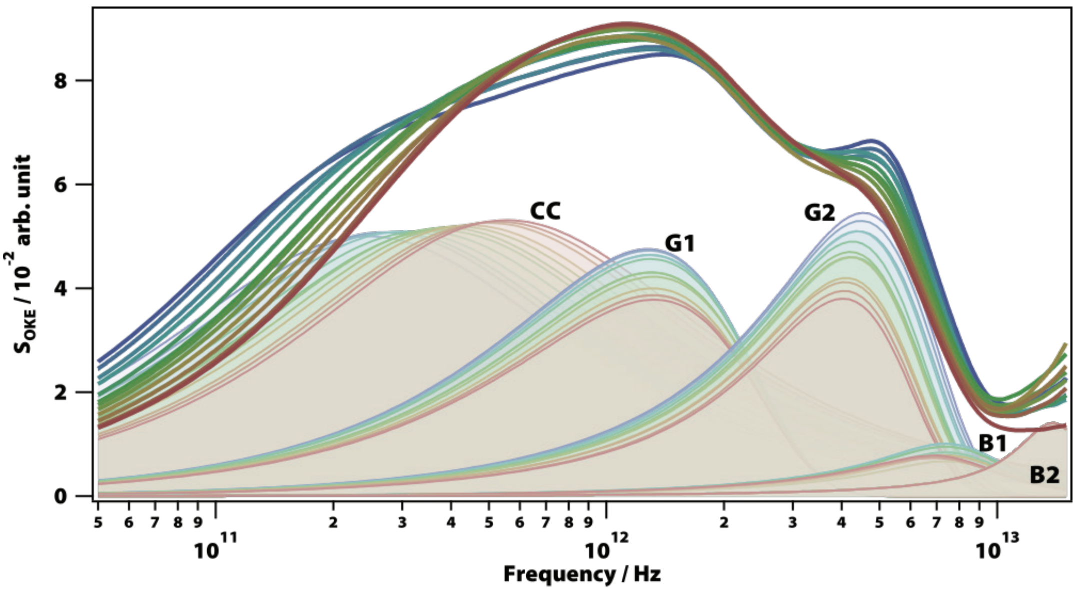

Supplementary Figure 2. *Experimental OKE spectra and the component fit-functions used to fit the data for water. The temperatures run from 298 (blue) to 353 (red) K in steps of 10 K. Each spectrum has been fitted using the combination of a Cole-Cole function (CC), two gaussian oscillators (G1, G2) and two brownian oscillators (B1, B2).*

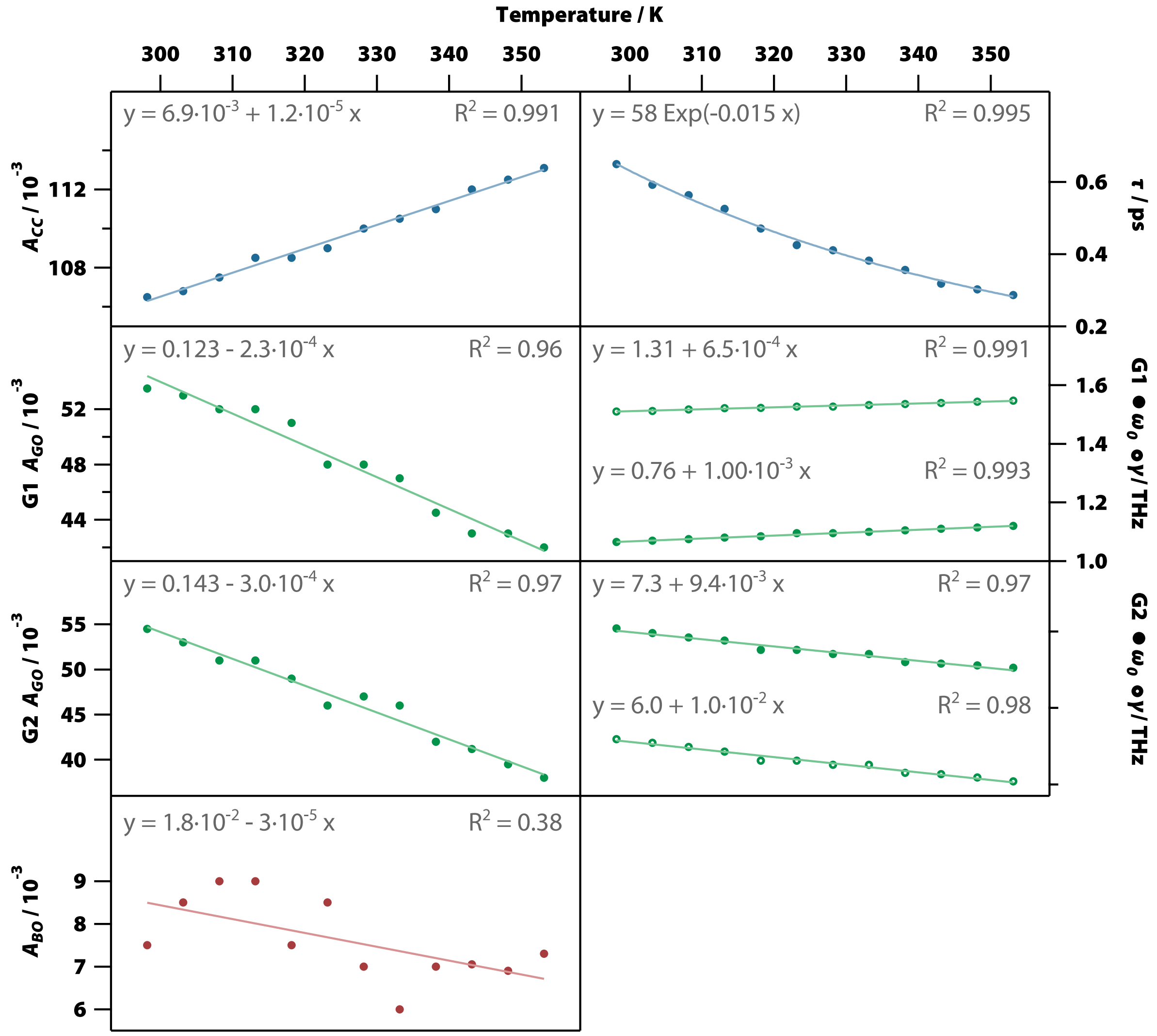

Supplementary Figure 3. *Influence of temperature on the fitted parameters of the functions used to model the water spectra.*

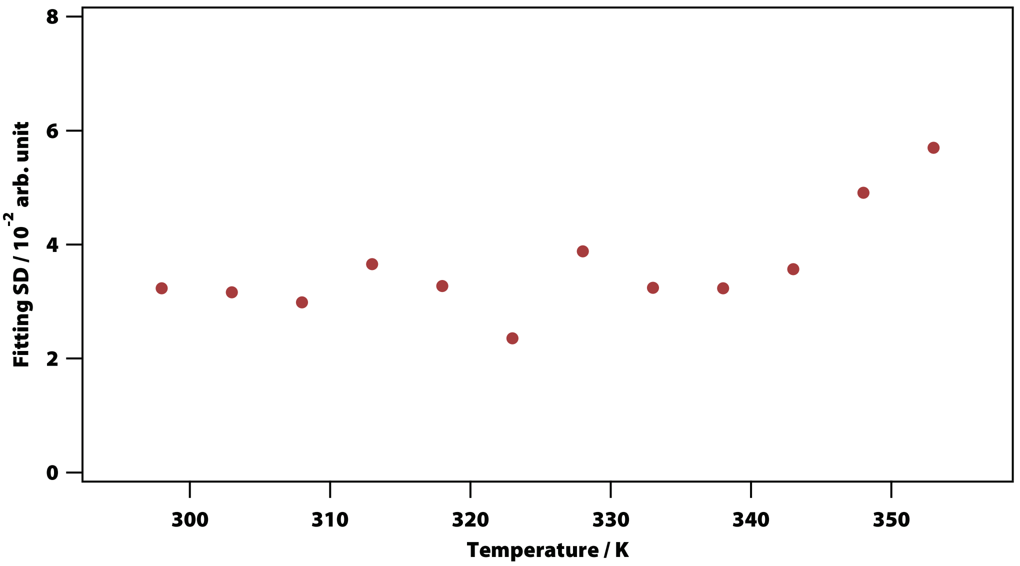

Supplementary Figure 4. *Standard deviation of the model from the measured OKE spectra of water at each temperature.*

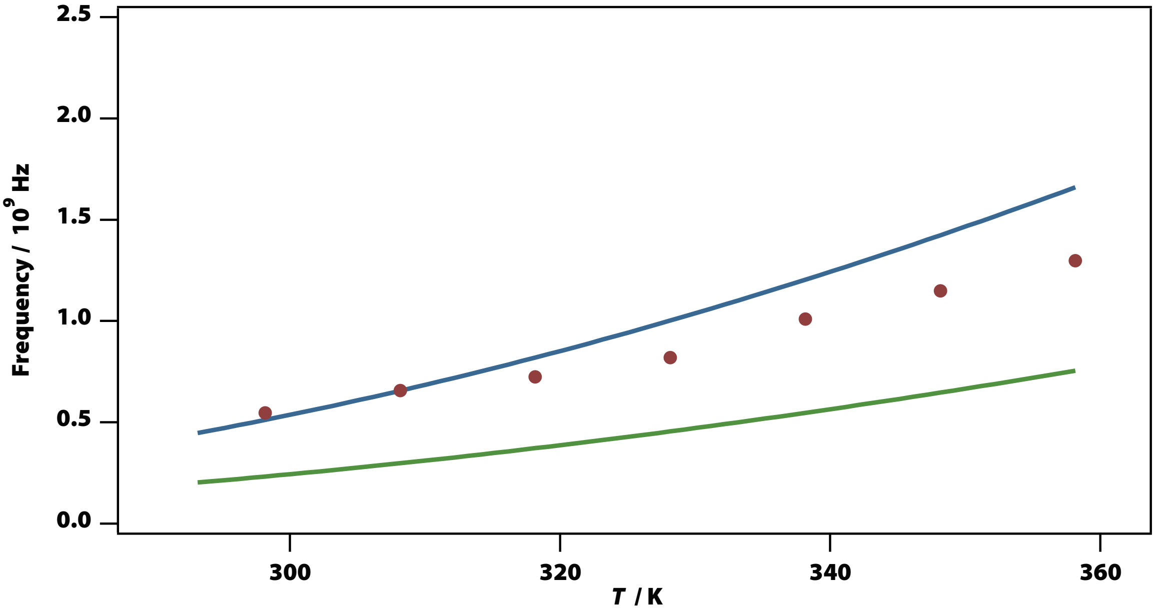

Supplementary Figure 5. *Estimated frequency of the rotational diffusion of a DNA 20mer for the mayor axis (blue) and minor axis (green). Red dots represent the measured frequency of the rotational diffusion in our OKE experiment.*

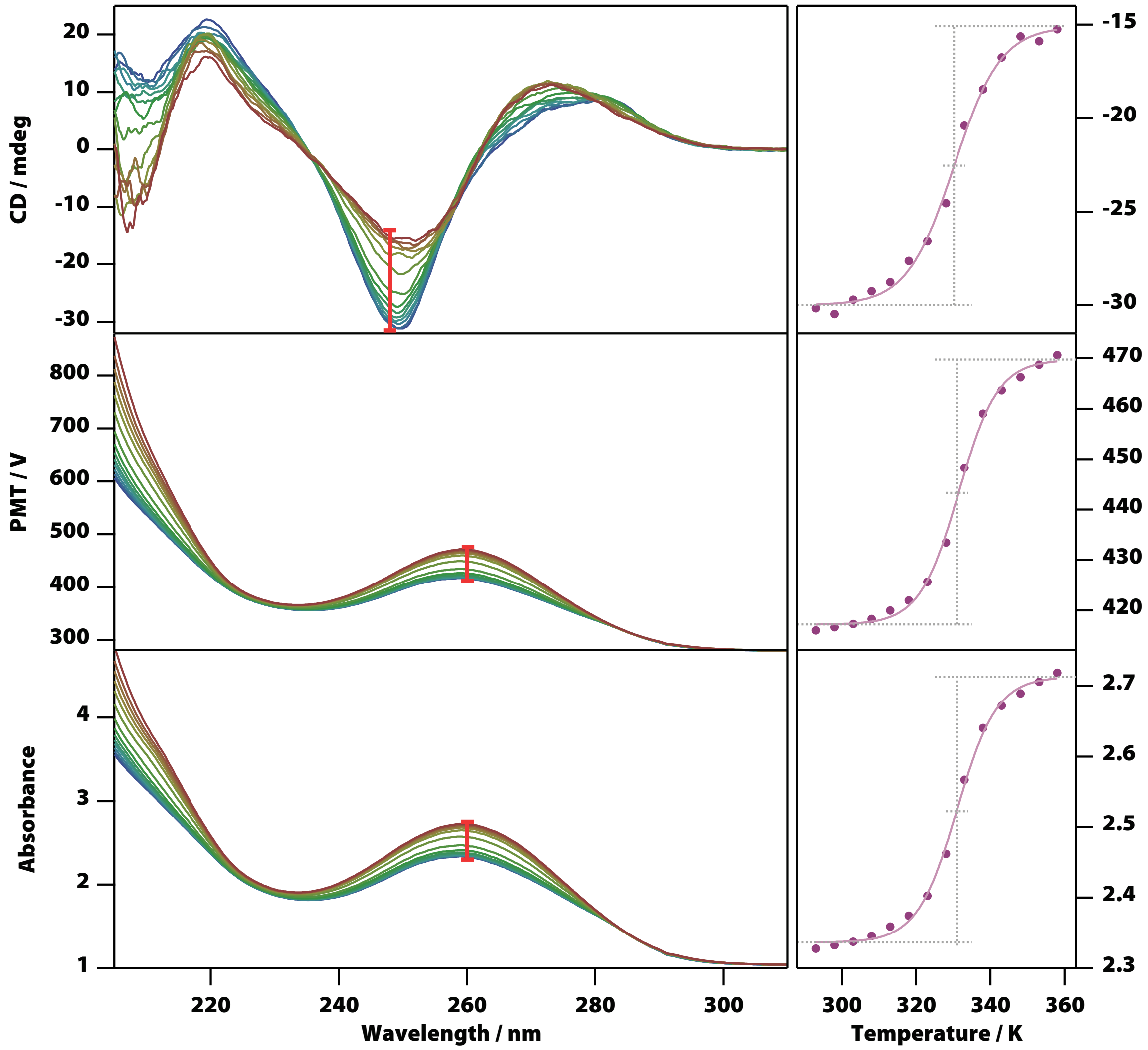

Supplementary Figure 6. *Thermal melting CD spectral profile of AT 20mer (293 to 358 K at intervals of 5 K). Red segments denote the wavelengths used to plot the melting curves on the right.*

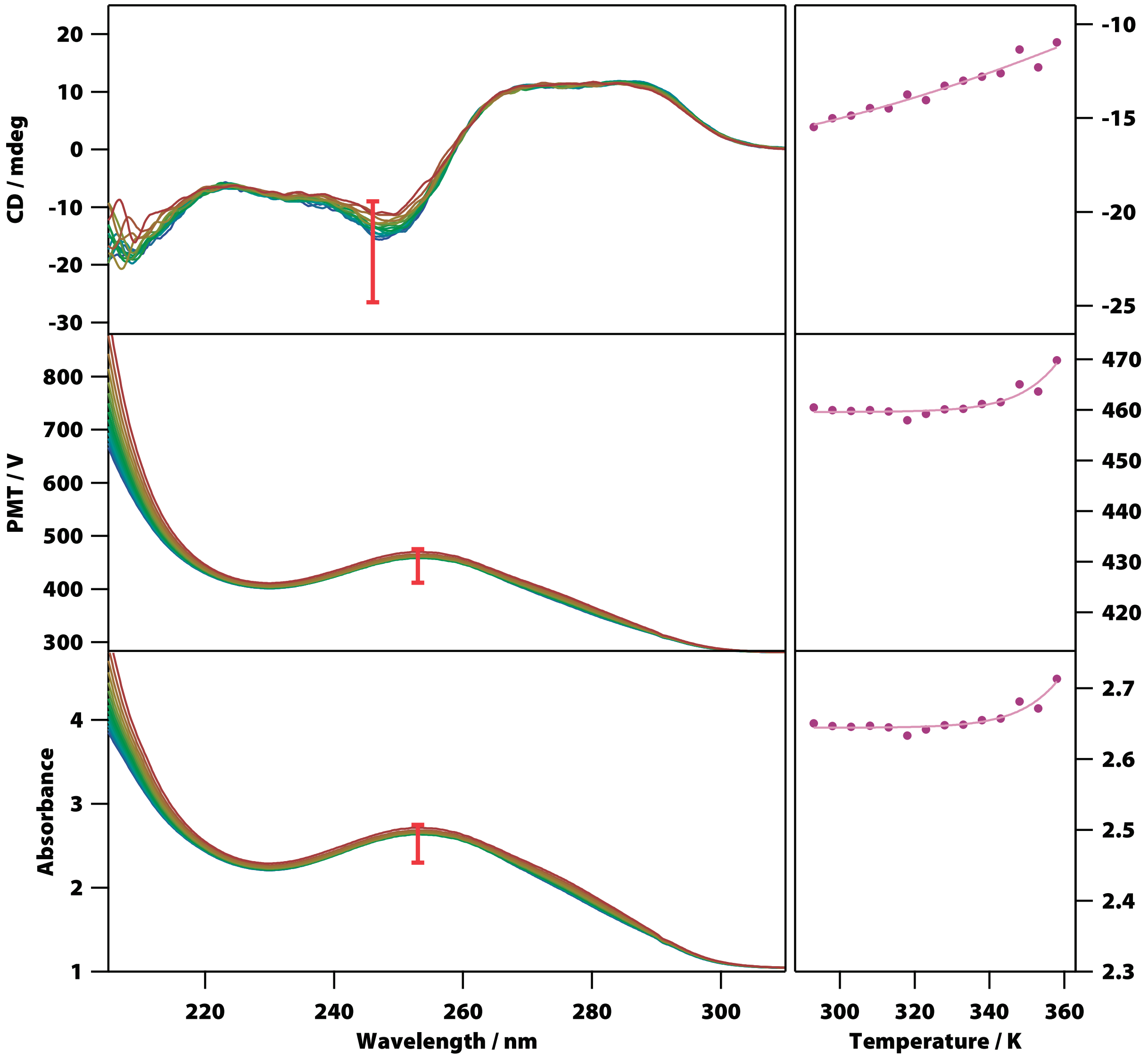

Supplementary Figure 7. *Thermal melting CD spectral profile of CG 20mer (293 to 358 K at intervals of 5 K). Red segments denote the wavelengths used to plot the melting curves on the right.*

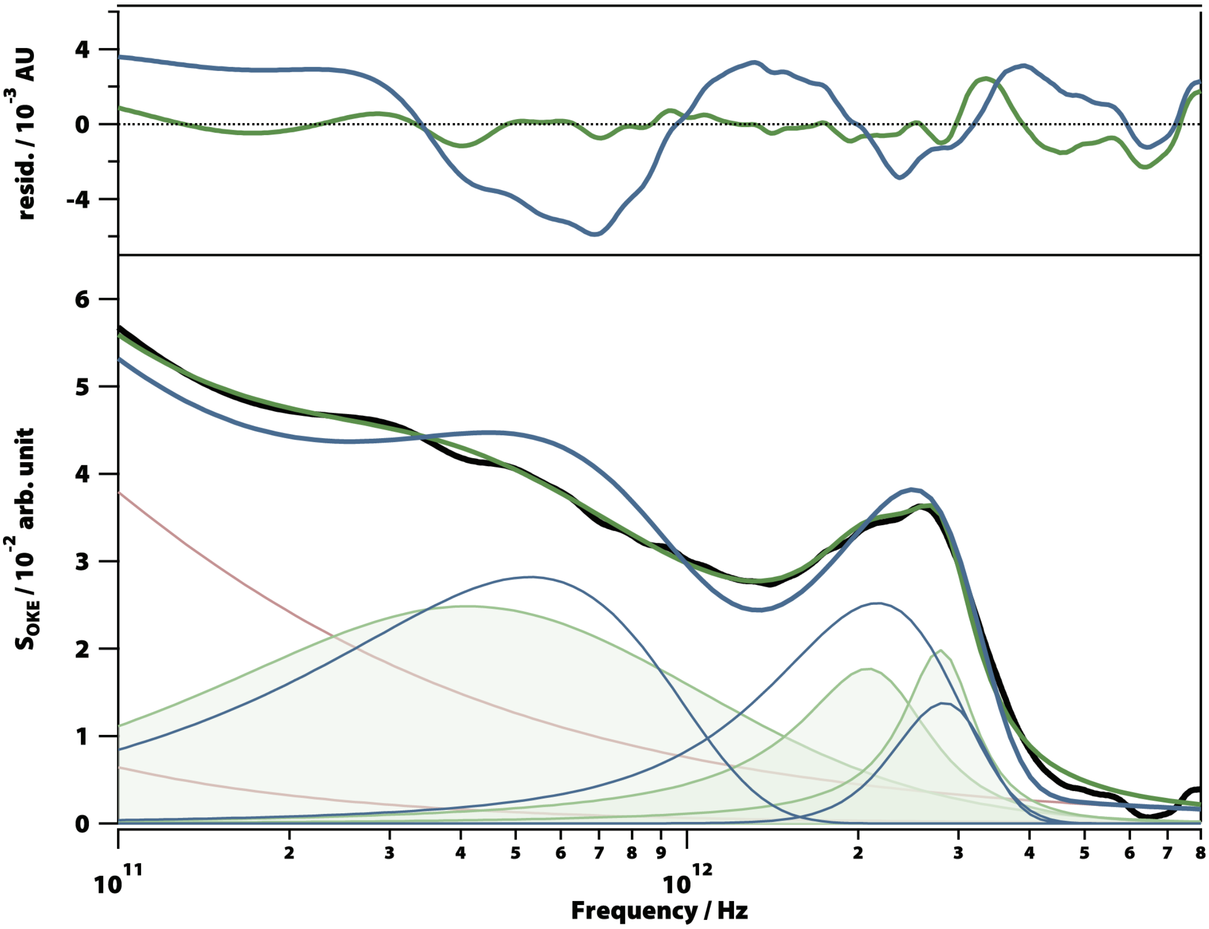

Supplementary Figure 8. *Experimental OKE spectrum of the 20 base-pair AT oligomer taken at 298 K and curve fits. Shown are the fits to the high-frequency portion of the spectrum that can be obtained using anti-symmetrised Gaussian functions (blue) or Brownian oscillator functions (green). Red lines represent the diffusional processes. The top panel shows the residuals of the fit, which are much larger for the fit using Gaussians.*

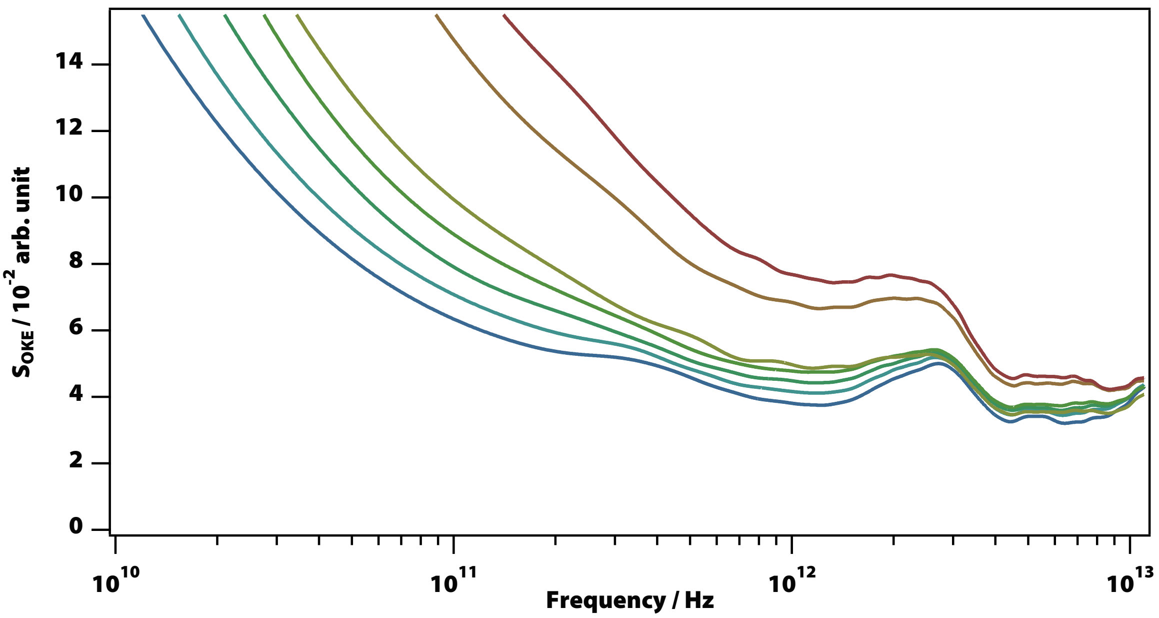

Supplementary Figure 9. *OKE spectra for CG 20mer at temperatures between 298 (blue) and 358 K (red) at intervals of 10 K.*

	Cole-Cole function (α = 0.96)		Gaussian oscillators						Brownian oscillator
			G1			G2			$\omega_o/2\pi$ = 8.3 THz $\gamma/2\pi$ = 3.92 THz
T / K	A_{CC}	τ / fs	A_G	ω_o /2π THz	γ / 2π THz	A_G	ω_o /2π	γ /2π THz	A_{BO}
298	0.1065	649	0.054	1.065	1.510	0.054	4.540	3.090	0.0075
303	0.1068	592	0.053	1.070	1512	0.053	4.480	3.045	0.0085
308	0.1075	563	0.052	1.075	1.517	0.051	4.420	2.990	0.0090
313	0.1085	525	0.052	1.080	1.521	0.051	4.380	2.930	0.0090
318	0.1085	471	0.051	1.085	1.522	0.049	4.260	2.810	0.0075
323	0.1090	424	0.048	1.095	1.527	0.046	4.260	2.810	0.0085
328	0.1100	410	0.048	1.095	1.527	0.047	4.205	2.755	0.0070
333	0.1105	381	0.047	1.100	1.532	0.046	4.205	2.755	0.0060
338	0.1110	356	0.044	1.105	1.535	0.042	4.100	2.650	0.0070
343	0.1120	318	0.043	1.110	1.539	0.041	4.080	2.635	0.0070
348	0.1125	302	0.043	1.115	1.543	0.040	4.056	2.590	0.0069
353	0.1131	286	0.042	1.120	1.547	0.038	4.026	2.540	0.0073

Supplementary Table 1. *Fitting parameters for water OKE spectra (Supplementary Figure 2).*

	Debye function		Cole-Cole function			Brownian oscillators			
						B1 $\omega_o/2\pi$ = 1.01 THz $\gamma/2\pi$ = 1.39 THz	B2 $\omega_o/2\pi$ = 1.38 THz $\gamma/2\pi$ = 0.75 THz	B3 $\omega_o/2\pi$ = 2.19 THz $\gamma/2\pi$ = 0.70 THz	B4 $\omega_o/2\pi$ = 2.83 THz $\gamma/2\pi$ = 0.50 THz
T / K	A_D	τ / ps	A_{CC}	τ / ps	α	A_{BO}	A_{BO}	A_{BO}	A_{BO}
298	0.94	234	0.22	11.1	0.77	0.042	0	0.011	0.007
308	1.05	191	0.25	10.6	0.76	0.044	0.003	0.0115	0.007
318	1.16	167	0.29	9.5	0.75	0.0415	0.006	0.0127	0.006
328	1.27	135	0.34	8.6	0.72	0.037	0.014	0.015	0.004
338	1.38	103	0.39	7.1	0.70	0.032	0.022	0.014	0.002
348	1.45	81	0.41	6.5	0.68	0.031	0.022	0.015	0.001
358	1.47	67	0.43	5.7	0.67	0.03	0.021	0.015	0.0005

Supplementary Table 2. *Fitting parameters for AT 20mer OKE spectra (Supplementary Figure 1)*

		13-mer	17-mer	13+17-mer
Debye function	A_D	0.9	1.3	1.1
	τ / ps	175	206	259
Cole-Cole function	A_{CC}	0.23	0.21	0.20
	τ / ps	11.1	8.7	11.1
	α	0.69	0.71	0.71
Brownian oscillator 1	A_{BO}	0.024	0.021	0.036
	ω_o / 2π THz	1.04	1.11	1.04
	γ / 2π THz	1.25	1.25	1.39
Brownian oscillator 2	A_{BO}	0.009	0.008	-
	ω_o / 2π THz	1.08	1.20	-
	γ / 2π THz	0.6	0.6	-
Brownian oscillator 3	A_{BO}	0.021	0.024	0.010
	ω_o / 2π THz	2.32	2.32	2.19
	γ / 2π THz	0.95	1.1	0.7
Brownian oscillator 4	A_{BO}	-	-	0.006
	ω_o / 2π THz	-	-	2.83
	γ / 2π THz	-	-	0.5

Supplementary Table 3. *Fitting parameters for complementary oligomers OKE spectra (Supplementary Figure 1)*

	Debye function 1		Debye function 2		Brownian oscillators B1 $\omega_o/2\pi$ = 1.48 THz $\gamma/2\pi$ = 1.08 THz	Brownian oscillators B2 $\omega_o/2\pi$ = 1.58 THz $\gamma/2\pi$ = 1.06 THz
[CMP] / M	A_D	τ / ps	A_D	τ / ps	A_{BO}	A_{BO}
0.1	0.12	7.9	0.01	1.1	0.003	0.008
0.2	0.25	16.0	0.05	2.2	0.012	0.016
0.3	0.34	16.7	0.05	2.2	0.018	0.022
0.4	0.48	18.3	0.06	1.9	0.026	0.031
0.6	0.72	27.8	0.10	2.7	0.034	0.041
0.8	0.98	39.8	0.15	3.5	0.047	0.052
1.0	1.25	58.9	0.19	4.1	0.053	0.061
1.2	1.47	65.2	0.20	3.9	0.061	0.074
1.6	2.00	94.4	0.21	4.6	0.066	0.084

Supplementary Table 4. *Fitting parameters for CMP OKE spectra (Supplementary Figure 1)*

Supplementary Note 1 – Water OKE spectra and subtraction procedure

Measured OKE spectra have contributions from the solvent and the solvated nucleic acid. In order to extract the latter, the spectrum of water was directly subtracted from the measured OKE spectra (Supplementary Figure 1, Supplementary Tables 1-4).[1] Water spectra are indistinguishable from the phosphate buffer spectra at the concentration employed in the experiments (0.15 M). To simplify the analysis, the spectra of water were simulated in the subtraction process using a model of the dependence of the water spectra on temperature. This model was created after the measurement and fitting of the OKE spectra of water between 298 and 343 K at intervals of 5 K (Supplementary Figure 2). Each spectrum was fitted using a combination of one Cole-Cole function for the diffusive translational motions of water molecules, two gaussian oscillators for the LA and TA phonon modes and two brownian oscillators for the librational modes[2,3] (Supplementary Table 1). Most of the librational modes of higher frequency (9 - 30 THz) can not be seen in the recorded OKE spectra but its contribution was fitted keeping the parameters of the brownian oscillator constant with temperature (A_{BO} = 0.007, $\omega_o/2\pi$ = 14.3 THz, and $\gamma/2\pi$ = 3.62 THz), since this band is scarcely affected by thermal variations in the temperature range of the experiments.[4]

Supplementary Figure 3 shows the influence of temperature on each parameter of the employed equations. Only the change on relaxation time with temperature could not be fitted with a linear equation and an exponential fitting had to be used. Most of the fittings show a good regression coefficient. In order to check its quality, the model was compared with the measured spectra and the standard deviation was calculated (Supplementary Figure 4). The low values of the standard deviations confirm that obtained equations are a good model of the water spectra.

Supplementary Note 2 – Rotational diffusion time estimations

The dimensions of a B-DNA 20-mer are 2.37 nm in diameter and 4.6 nm in length.[5] The two rotational frictional drag coefficients, modelling the molecule as an ellipsoid, are for the major axis

$$f_M = \frac{16}{3}\pi\eta a b^2 \tag{1}$$

and for the minor axis[6,7]

$$f_m = \frac{8\pi\eta a^3/3}{\ln\frac{2a}{b} - \frac{1}{2}}, \tag{2}$$

where a is the radius of the major axis and b is the radius of the minor axis, and η is the shear viscosity of the medium. Using a = 2.3 nm, b = 1.19 nm, and the following expression[8]

$$\eta = 2.414\times10^{-5}\times10^{247.8/(T-140)} \tag{3}$$

approximating the experimental shear viscosity of liquid water, where T is the temperature in Kelvin, to calculate the viscosity, we obtain $f_M = 4.82\times10^{-29}$ kg m^2 s^{-1} and $f_m = 1.06\times10^{-28}$ kg m^2 s^{-1} at 298 K and $f_M = 1.79\times10^{-29}$ kg m^2 s^{-1} and $f_m = 3.93\times10^{-29}$ kg m^2 s^{-1} at 358 K. The orientational diffusion coefficient is then given by[9]

$$D_r = \frac{kT}{f}, \tag{4}$$

where k is Boltzmann constant. Our experiment has been made between 298 and 358 K, so at these temperatures the values of the rotational coefficients are $D_M = 8.54\times10^7$ Hz and $D_m = 3.88\times10^7$ Hz at 298 K and $D_M = 2.76\times10^8$ Hz and $D_m = 1.26\times10^8$ Hz at 358 K.

As OKE is a four-wave mixing technique, the orientational relaxation time constant is given by[9]

$$\tau = \frac{1}{6D} \ . \tag{5}$$

Thus, the orientational relaxation time constants for our oligomer are τ_M = 2.00 ns and τ_m = 4.40 ns at 298 K and τ_M = 0.60 ns and τ_m = 1.25 ns at 358 K.

Supplementary Figure 5 shows the dependence on temperature of the fitted relaxation frequency of the band associated with the orientational diffusion of our AT oligomer. Also shown are the frequency values predicted by the SED equation obtained from the inverse of the orientational relaxation time $\nu = \tau^{-1}$.

Supplementary Note 3 – Circular dichroism spectra

The melting temperature of the oligomers studied in the article has been determined by tracking the changes in the conformation of the nucleic acids with temperature using circular dichroism and absorption spectroscopies. CD and absorption spectra of a 100-μM solution of oligomer in a 0.1 cm pathlength quartz cuvette have been recorded at intervals of 5 K between 293 and 358 K in a JASCO J-810 spectropolarimeter with a thermoelectric temperature control system (± 0.1 K). The changes in the spectra with temperature have been plotted and fitted to sigmoid curves. The melting point of the oligomer is taken as the temperature at which the sigmoid curve reaches half of its maximum.

AT 20mer

The change in the degree of ellipticity of the sample at 246 nm shows a melting point for AT 20mer of 330.1 ± 0.6 K (see Supplementary Figure 6). These are the data used in the Figure 1 of the article. The maximum of the absorption band at 260 nm has been used to determine the melting point of the AT 20mer, obtaining a T_m = 331.0 ± 0.4 K

CG 20mer

The absorption and CD spectra for CG 20mer only show significant changes above 343 K (see Supplementary Figure 7), so this oligomer does not melt at the temperatures accessible in the OKE experiments. From the curve drawn with the absorption data, a hypothetical melting point of 382 K can be estimated.

Supplementary Note 4 – Nonlinear curve-fitting procedures

Fit functions

Standard fit functions were used to fit to the data. Diffusional processes are modelled using the Havriliak-Negami function

$$S_{HN}(\omega) = \frac{A_{HN}}{\left[1+(i\omega\tau)^{\alpha}\right]^{\beta}} \ , \tag{6}$$

where A_{HN} is the amplitude of the function, α and β are empirical parameters, ω is the angular frequency, and τ is the relaxation time of the diffusional process. This function reduces to the Debye function for $\alpha = \beta = 1$, which is commonly used to model the lowest frequency orientational diffusion band. The function reduces to the Cole-Cole function for β = 1, which is often used to model intermediate frequency relaxational processes. In particular, we have shown that the lowest frequency part of the OKE spectrum of liquid water can be modelled using a Cole-Cole function with α = 0.96.

Peaks in the spectrum due to underdamped librational and vibrational modes were modelled using the Brownian oscillator function[10]

$$S_{BO}(\omega) = \frac{A_{BO}\omega_0^2}{\omega_0^2 - \omega(\omega + 2i\gamma)} \ , \tag{7}$$

where A_{BO} is the amplitude parameter, ω_o is the undamped oscillator angular frequency, and γ is the damping rate, that has units of angular frequency.

The phonon modes observed in water spectra were fitted using the anti-symmetrised Gaussian function

$$S_G(\omega) = A_G \left\{ \exp\left[-\frac{(\omega-\omega_0)^2}{\gamma^2} \right] - \exp\left[-\frac{(\omega+\omega_0)^2}{\gamma^2} \right] \right\}, \quad (8)$$

where A_G is the amplitude parameter, ω_o is the undamped oscillator angular frequency, and γ is the damping rate.

Fitting to anti-symmetrised Gaussian vs. Brownian oscillator functions

Supplementary Figure 8 shows a representative experimental OKE spectrum of the 20 base-pair AT oligomer taken at 298 K. As described in the main text, the high frequency region (0.1-5 THz) contains a number of bands, two of which have been assigned to phonon-like delocalised modes. In previous work, we have studied such bands in liquids and found that they could be modelled well using anti-symmetrised Gaussian functions suggesting an inhomogeneous distribution of environments.[3,11-13] Here, we attempted to fit the high-frequency OKE spectrum of the 20 base-pair AT oligomer with either anti-symmetrised Gaussian functions (Supplementary Equation (8)) or Brownian oscillator functions (Supplementary Equation (7)). As can be seen in Supplementary Figure 8, the fit to Brownian oscillator functions is far superior. We judge the goodness of the fit using the χ^2 value, which is estimated at χ^2 = 1.36 for the fit using Brownian oscillator functions and χ^2 = 16.0 for the fit using anti-symmetrised Gaussian functions. Although experiments such as spontaneous Raman scattering and related techniques such as OKE cannot distinguish between homogeneous and inhomogeneous broadening,[14-16] the Brownian oscillator function is usually considered proof of a homogeneously broadened line. In the unlikely case that an inhomogeneous distribution gave rise to a Lorentzian (Brownian) lineshape, we can still state that the distribution is narrow with the width much less than the average frequency.

Supplementary note 5 – CG 20mer OKE Spectra

The OKE spectra of the CG 20mer (see Supplementary Figure 9) are analogous to the AT 20mer spectra and also are in accordance with the data obtained with circular dichroism spectroscopy. The only changes with increasing temperature in the OKE spectra correspond to the shifting of the molecular orientational diffusion band to higher frequencies.

The OKE spectra of the CG 20mer also clearly show a band with maximum at 2.7 THz like the band B4 of the AT 20mer OKE spectra (Figure 1). The behaviour of the band with increasing temperature supports the association of the band with the double strand conformation of the oligomer, since the band only starts changing its shape at the higher temperatures of the experiment.

Supplementary References

1 Turton, D. A. *et al.* Terahertz underdamped vibrational motion governs protein-ligand binding in solution. *Nat. Chem.* **5**, 3999 (2014).

2 Turton, D. A. *et al.* The structure and terahertz dynamics of water confined in nanoscale pools in salt solutions. *Faraday Disc.* **150**, 493-504 (2011).

3 Turton, D. A., Corsaro, C., Martin, D. F., Mallamace, F. & Wynne, K. The dynamic crossover in water does not require bulk water. *Phys. Chem. Chem. Phys.* **14**, 8067-8073 (2012).

4 Sassi, P. *et al.* Volume properties and spectroscopy: A terahertz Raman investigation of hen egg white lysozyme. *J. Chem. Phys.* **139**, 225101 (2013).

5 Dickerson, R. E., Drew, H. R., Conner, B. N., Kopka, M. L. & Pjura, P. E. Helix Geometry and Hydration in A-DNA, B-DNA, and Z-DNA. *Cold Spring Harb Symp Quant Biol* **47**, 13-24 (1983).

6 Berg, H. C. *Random walks in biology*. 2 edn, (Princeton University Press, 1983).

7 Perrin, F. Mouvement Brownien d'un ellipsoïde (I). Dispersion diélectrique pour des molécules ellipsoïdales. *Le Journal de Physique et le Radium* **5**, 497-511 (1934).

8 Al-Shemmeri, T. *Engineering Fluid Mechanics*. (Ventus Publishing, 2012).

9 Turton, D. A. & Wynne, K. Stokes–Einstein–Debye Failure in Molecular Orientational Diffusion: Exception or Rule? *J. Phys. Chem. B* **118**, 4600-4604 (2014).
10 Cho, M., Du, M., Scherer, N., Fleming, G. & Mukamel, S. Off-resonant transient birefringence in liquids. *J. Chem. Phys.* **99**, 2410-2428 (1993).
11 Sonnleitner, T. *et al.* Ultra-Broadband Dielectric and Optical Kerr-Effect Study of the Ionic Liquids Ethyl and Propylammonium Nitrate. *J. Phys. Chem. B* **119**, 8826-8841 (2015).
12 Turton, D. A., Martin, D. F. & Wynne, K. Optical Kerr-effect study of trans- and cis-1,2-dichloroethene: liquid-liquid transition or super-Arrhenius relaxation. *Phys. Chem. Chem. Phys.* **12**, 4191-4200 (2010).
13 Turton, D. A. *et al.* Structure and dynamics in protic ionic liquids: A combined optical Kerr-effect and dielectric relaxation spectroscopy study. *Faraday Disc.* **154**, 145-153 (2012).
14 Loring, R. F. & Mukamel, S. Selectivity in coherent transient Raman measurements of vibrational dephasing in liquids. *J. Chem. Phys.* **83**, 2116-2128 (1985).
15 Muller, M., Wynne, K. & Vanvoorst, J. The Interpretation of Echo Experiments. *Chem. Phys.* **125**, 225-230 (1988).
16 Wynne, K., Muller, M., Brandt, D. & Vanvoorst, J. Diagrammatic Density-Matrix Analysis of the Raman Photon-Echo. *Chem. Phys.* **125**, 211-223 (1988).
